# Eye–head coordination during rapid gaze shifts in soccer scanning

**DOI:** 10.7717/peerj.21779

**Published:** 2026-10-22

**Authors:** Junki Saito, Tomohiro Kizuka, Seiji Ono

**Affiliations:** 1 Graduate School of Comprehensive Human Sciences, University of Tsukuba, Tsukuba, Ibaraki, Japan; 2 Institute of Health and Sport Sciences, University of Tsukuba, Tsukuba, Ibaraki, Japan; 3 Advanced Research Initiative for Human High Performance (ARIHHP), University of Tsukuba, Tsukuba, Ibaraki, Japan

**Keywords:** Soccer, Scanning, Eye–head coordination, Gaze shifts, Eye movements, Visual strategy

## Abstract

**Background:**

The visual exploratory behavior involving rapid gaze shifts that supports cognitive processes is called “scanning”. Scanning in soccer is a foundational behavior that enables players to explore their environment, supporting rapid and accurate situational judgments. However, since most previous studies have focused on head movements, the eye–head coordination underlying gaze shifts is insufficiently understood. This study aimed to determine the coordinated roles of eye and head movements during gaze shifts in soccer scanning.

**Methods:**

Twenty male collegiate soccer players performed a passing task paired with video-based situational judgments while eye and head movements were recorded using an eye tracker system with a built-in gyroscope. We detected eye and head velocities and amplitudes during rapid gaze shifts.

**Results:**

As a result, peak eye velocity was significantly larger than peak head velocity (*d* = 4.09, *p* < 0.001). The cross-correlation (CC) between gaze and eye velocities (0.92) was significantly greater than that between gaze and head velocities (0.58). Furthermore, head contribution increased with larger gaze-shift amplitudes and under higher temporal constraints. These results indicate that gaze shifts are characterized by a predominant contribution of eye movements, while head movements provide task-dependent adjustments to the overall gaze behavior. In addition, eye and head velocities were negatively correlated (*r* =  − 0.60, *p* = 0.005), indicating individual differences in eye–head coordination tendencies.

**Conclusion:**

Our findings suggest that eye movements serve as the primary effector during gaze shifts in soccer scanning, showing consistent kinematic characteristics across varying task demands. Furthermore, the head could contribute in a complementary and adaptive manner, increasing its involvement in response to movement demands or situational constraints.

## Introduction

In sports such as soccer, where play rapidly alternates between attack and defense within a wide field of view, the quality of cognitive processing is a critical determinant of performance (Davids, Williams & Williams, 2005; Mann et al., 2007). Visual exploratory behaviors such as gaze shifts involving head movements that support cognitive processes are referred to as “scanning” (Jordet, 2005; McGuckian et al., 2020). It is known that greater frequency and quality of scanning enable players to anticipate more information and gain a competitive advantage. Indeed, observational studies have shown that scanning is a foundational behavior supporting situational judgments (Aksum et al., 2021a). Furthermore, recent studies at international soccer tournaments have quantified head direction (Pokolm et al., 2023) and scanning frequency (Pokolm et al., 2022), providing the importance of scanning as an essential component for performance.

Previous research on scanning, while emphasizing its importance, has primarily focused on the frequency of head movements and the characteristics of fixations. For example, in soccer, the number and timing of head turns have repeatedly been reported to be associated with performance (Jordet, 2005; McGuckian et al., 2020; Jordet et al., 2020). In addition, the duration and location of fixations are known to influence the accuracy of situational judgments (Roca et al., 2011; Vítor De Assis et al., 2021). Moreover, visual exploratory behavior has been shown to support tactical decision-making even in youth players (Klatt & Smeeton, 2022). These findings provide strong support for the importance of scanning. However, many previous studies have used head turn frequency as a proxy measure of scanning (Aksum et al., 2021b; McGuckian et al., 2019). Studies that have simultaneously measured eye movements are extremely rare (Van Biemen & Mann, 2024).

Another study focusing on fixations has emphasized static gaze locations “where one looks” rather than sufficiently addressing the dynamic aspects of gaze shifts and their coordination structure. Recent reviews have also pointed out that although advances in eye-tracking technology have increased laboratory-based studies, knowledge of ocular dynamics under ecologically valid competitive conditions remains lacking (Kredel et al., 2023). Furthermore, even comprehensive reviews addressing the relationship between decision-making and visual exploratory behavior have not explicitly examined the coordination structure of gaze shifts (Silva, Conte & Clemente, 2020).

Gaze shifts are a foundational element of scanning, enabling players to efficiently acquire diverse environmental information and providing the prerequisite basis for subsequent decision-making (Roca, Williams & Ford, 2014; Hüttermann, Noël & Memmert, 2018). Aksum et al. (2021b) defined in-match scanning as “active head and eye movements performed with the intention of momentarily averting one’s gaze from the ball to search for information about teammates, opponents, referees, or spaces relevant to the unfolding play”. This definition implies that gaze shifts, momentarily diverting the eyes from the ball for information gathering, are indispensable components of scanning. Furthermore, studies in junior and youth players have reported that the efficiency of exploratory patterns directly influences action selection (Klatt & Smeeton, 2022; Vítor De Assis et al., 2021). Exploratory activity, including gaze shifts, has also been demonstrated as a core factor supporting the accuracy and efficiency of decision-making (Mann et al., 2007), with attentional flexibility identified as one of the underlying cognitive abilities (Hüttermann, Noël & Memmert, 2018).

In contrast to practical research, basic research has examined the mechanisms of eye–head coordination during gaze shifts in detail, reporting changes in ocular and head contributions as a function of gaze amplitude (Freedman, 2008), individual differences (Fuller, 1992), and physiological constraints of ocular motion (Bahill, Clark & Stark, 1975; Zangemeister, Jones & Stark, 1981). While one applied study has examined eye–head coordination in referees’ on-field visual exploration (Van Biemen et al., 2023), investigations probing angular-velocity profiles and qualitative aspects of coordination remain uncertain. Thus, it is still unclear how mechanisms of gaze control established in basic research manifest in the practical context of scanning in soccer, particularly under its highly dynamic and complex environmental demands.

Based on these considerations, the coordination structure of eye–head contributions underlying gaze shifts, the foundational component of scanning, has not yet been systematically examined in applied sport contexts. The next step in scanning research is not only to examine where players direct their gaze or how often the head turns, but to clarify how the gaze is shifted through the coordinated contributions of eye and head movements. This approach allows for the systematic clarification of the functional role of the eye and head within the context of soccer scanning. Therefore, the present study aimed to quantitatively elucidate the structural and functional characteristics of eye–head coordination during gaze shift in soccer scanning. To achieve this objective, we analyzed the coordination system from three complementary perspectives aligned with the multi-structural nature of scanning: (1) structural contributions of eye and head movements to gaze formation, (2) coordination relationships between their velocity profiles, and (3) functional roles expressed through movement variability and conditional adjustments.

## Materials & Methods

### Participants

Portions of this text were previously published as part of a preprint (https://doi.org/10.1101/2025.09.19.677484).

Participants were 20 male university soccer players (mean age: 19.8 ± 1.4 years; height: 171.4 ± 6.7 cm; body mass: 66.8 ± 7.5 kg; playing experience: 13.7 ± 2.8 years). All participants were right-foot dominant, had normal or corrected-to-normal vision, and reported no motor impairments. Many of the participants were regular starters on teams competing in the Kanto University Soccer League and possessed a highly competitive level. Participants trained approximately 6 days per week, with sessions lasting approximately 2 h per day. Positional distribution included ten central midfielders or attacking midfielders, nine wide players (side backs or side midfielders), and one forward. An *a priori* power analysis was conducted using G*Power (Faul et al., 2007). The assumed effect size (Cohen’s *d* = 0.65–0.70) was based on medium-to-large effect sizes reported in previous studies on gaze behavior and eye–head coordination (*e.g.*, Hintermann et al., 2025; Bickmann et al., 2020), and the analysis was conducted with reference to the primary outcome measures related to gaze-shift kinematics. Based on this effect size, *α* = 0.05, and statistical power of 0.80, the required sample size was estimated to be 18–20 participants. The final sample size of 20 participants, therefore, met the requirement for sufficient statistical power. Furthermore, the selected sample size is consistent with the typical range reported in eye-tracking research in sports science (Kredel et al., 2023). The study was conducted in accordance with the Declaration of Helsinki (2013), and all experimental protocols were approved by the Research Ethics Committee of the Institute of Health and Sport Sciences, University of Tsukuba (Approval number: 025-4). Prior to participation, all participants were informed of the study’s purpose and procedures and provided written informed consent.

### Procedure

In this study, we used an experimental task simulating a soccer passing situation to examine the relationship between head and eye movements and gaze shift strategies during scanning (Fig. 1). Participants stood at a passing location 2.4 m in front of a large screen (280 × 480 cm; Fig. 1-① and ②) and executed a one-touch pass toward a ball (Mikasa FT-550b FPQ) released from a ramp placed 90° to their left (custom-made apparatus: L-shaped angle; Fig. 1-③). Simultaneously with the ball becoming visible, a 2-*versus*-2 offensive–defensive video was projected onto the screen (EPSON EB-1771W; Fig. 1-④), and participants selected the pass direction toward the side displaying an unmarked teammate. Each participant completed 102 trials, comprising two temporal conditions × 17 trials × 3 blocks. After release, the ball was temporarily occluded by a custom-made visual shield (L-shaped angle with plastic board; Fig. 1-⑤) on the ramp and reappeared at a position three m before the passing point. An infrared sensor (Arduino Uno R3; Fig. 1-⑥) was placed at the reappearance point, triggering the projection of the situational judgment task on the screen as the ball passed.

**Figure 1:**
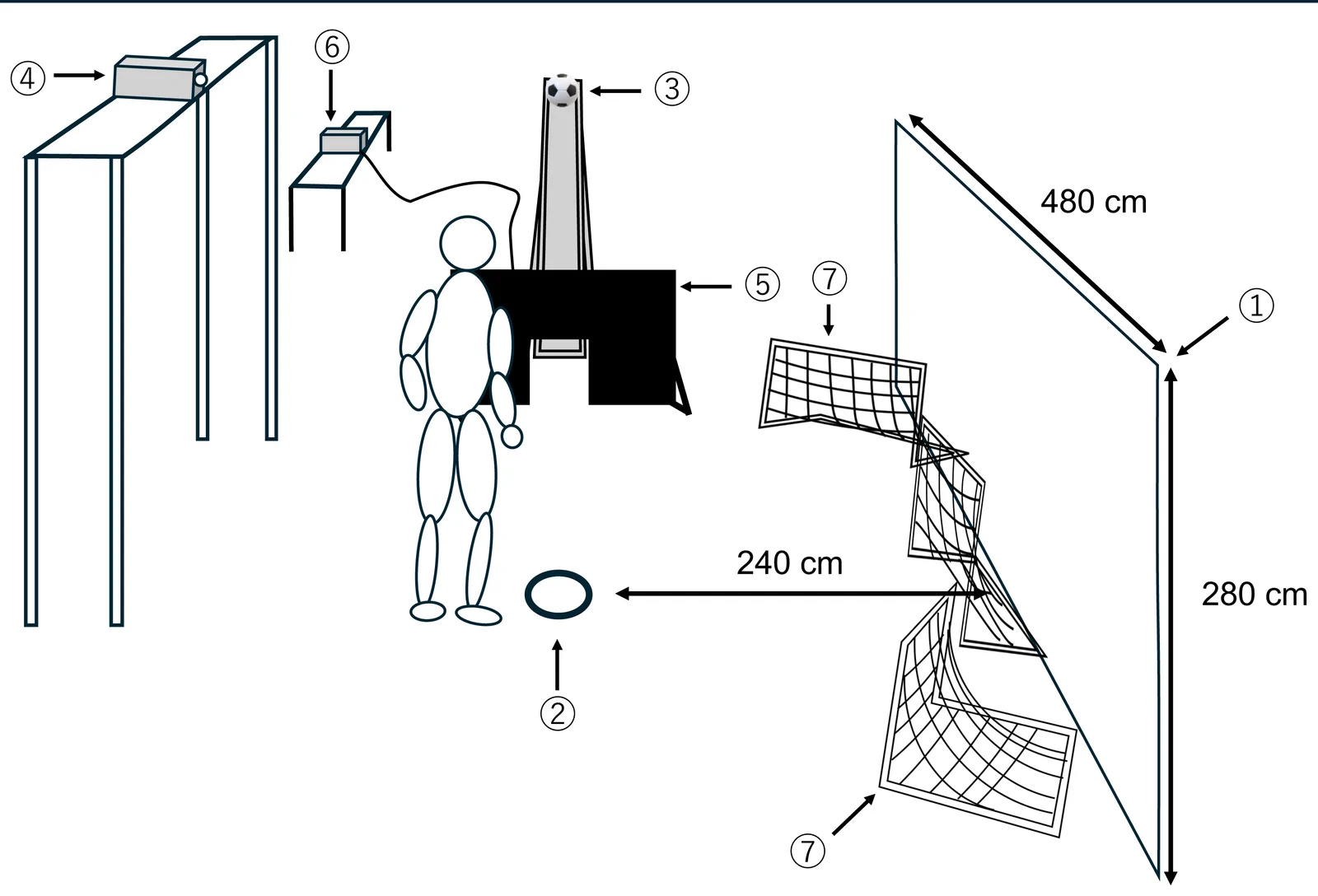
Overview of the experimental setup. (1) Screen; (2) Passing location; (3) Ball-release ramp; (4) Projector; (5) Occlusion screen; (6) Infrared sensor; (7) small target goals.

The target player appeared in one of three possible directions, left, center, or right, along with an additional dummy condition in which no free player was displayed. Participants were instructed to pass the ball toward small target goals (custom-made apparatus; Fig.  1-⑦) positioned in three directions (left, center, and right), corresponding to the location of the unmarked teammate. A total of 48 video clips (randomized player movements) were used, with 42 clips presented twice each and the remaining six clips presented once, plus four dummy clips, each repeated 3 times, resulting in 102 trials in total. The large screen was positioned 2.4 m in front of participants, yielding a horizontal visual angle of approximately 90°. The ramp (ball launch) and the screen were positioned perpendicularly (90° apart), requiring participants to perform relatively large horizontal gaze shifts (yaw rotations) involving both the eye and head to locate the screen and identify the target players. Note that since this study focused on eye–head coordination during the initial scanning phase of gaze shift, gaze behaviors associated with situational judgment on the screen were not included in the analysis. The two temporal conditions (800 ms and 1,600 ms) were introduced to examine whether the fundamental eye–head coordination structure is preserved under different time constraints. The 800 ms and 1,600 ms conditions were defined as the duration from when the ball passed the reappearance point to when it reaching the kicking position. These two durations were achieved by adjusting the starting height of the ball on the release ramp: releasing the ball from a higher point increased its acceleration and resulted in an arrival time of approximately 800 ms, whereas releasing it from a lower point reduced acceleration and produced an arrival time of approximately 1,600 ms. In both conditions, the moment the ball passed the infrared sensor automatically triggered the presentation of the situational judgment stimulus, ensuring precise timing control across trials. These durations represented the available time for participants to shift their gaze, make situational judgments and prepare their passing movement. In football, perceptual–motor behavior is known to be modulated by temporal pressure, with shorter available time inducing more constrained and anticipatory visual exploration (Vater, Williams & Hossner, 2020). Therefore, introducing two temporal conditions enabled us to examine whether and how gaze shift dynamics and eye–head coordination adjust under varying time constraints.

The specific durations (800 ms and 1,600 ms) were determined based on pilot testing, which ensured both ecological validity and appropriate task difficulty for university-level soccer players. This setup reproduced contextual features of actual soccer, including “anticipation of pass-impact timing” and “maintenance of ball gaze at the moment of impact” (Van Biemen & Mann, 2024), as well as “situational judgments under time pressure”. Participants were instructed to maintain their gaze straight toward the ramp while the ball was occluded, and then to initiate a gaze shift toward the screen once the ball reappeared, as illustrated in Fig. 1. A photograph of the full experimental setup, including the participant, eye-tracking device, ball-release ramp, and projection screen, is provided in supplemental file to facilitate visualization of the spatial arrangement and apparatus configuration. While the initial head and eye orientation was standardized through the fixation instruction and objective exclusion criterion, movement during the gaze shift phase was not mechanically constrained and was left to each participant. Foot placement and trunk rotation were not controlled during the task, and trunk co-rotation may potentially contribute to the head-in-space measures recorded by the gyroscope.

### Apparatus

Eye movements during the task were recorded using an eye-tracking device (Pupil Invisible glasses; Pupil Labs, Berlin, Germany). The sampling rates of the eye cameras and the scene camera were 200 Hz and 30 Hz, respectively, and eye-position data were acquired at 200 Hz. Head movements were recorded with the device’s built-in gyroscope at a sampling rate of 200 Hz. Prior to data collection, a single-point offset correction was performed as a calibration using the Pupil Invisible Companion App (the dedicated smartphone application for the device) by instructing the participant to fixate on a predefined target. Furthermore, a verification procedure was conducted before each session. Participants fixated on eight known spatial landmarks (the four corners of the screen and the four corners of the small target goals), and gaze alignment with these targets was visually confirmed in real time by the experimenter. This verification followed the manufacturer’s recommended offset correction procedure for the Pupil Invisible system, in which the experimenter manually adjusted gaze alignment using the Companion App. The acceptance criterion was to visually confirm that the gaze corresponded to each target served. The device has been reported to provide relatively high gaze accuracy under naturalistic conditions (mean error ∼1.1°; Hooge et al., 2024). Throughout the experiment, the experimenter continuously monitored gaze data and device placement, and adjustments were made as needed to maintain proper fit. Trials in which gaze data could not be obtained due to device misalignment or inadequate device placement were excluded; no such cases occurred during the experiment.

### Data processing

Data were recorded using Pupil Invisible (Pupil Labs) and downloaded to Pupil Player *via* Pupil Cloud, after which eye movement and gyroscope (Inertial Measurement Unit (IMU)) data were exported in CSV format. Both data streams were recorded at 200 Hz and synchronized based on a common timestamp. All data were processed offline after the recording session. Eye position data were obtained as normalized pixel coordinates (0–1 range) and converted to visual angles (degrees) by linearly scaling according to the device’s field of view (horizontal: 82° corresponding to 1088 pixels; vertical: 82° corresponding to 1080 pixels). The coordinate origin of the scene camera was set at the center of the screen. Specifically, visual angle (°) was calculated as (*p* − 0.5) × 82, where *p* denotes the normalized pixel coordinate (0–1 range). This linear approximation is appropriate given that the analyses focused on relative changes in angular velocity and position rather than absolute spatial localization. To model the coordination between eye and head movements, we defined each movement within its respective coordinate frame and employed an additive approximation model to integrate them. Specifically, three coordinate systems were distinguished: head-in-space, eye-in-head, and eye-in-space (gaze). In this framework, gaze was modeled as the additive combination of eye-in-head and head-in-space rotations, and the gaze signal was calculated as the sum of these two components, as previous studies using the same parameters (Freedman, 2008; Kishita, Ueda & Kashino, 2020; Schröder et al., 2021). Since this simplified kinematic model has been widely adopted to quantify the relative contributions of the eye and head in the horizontal plane, it was considered sufficiently accurate for characterizing the coordination structure during gaze shifts. Furthermore, gaze data in this study were used not for spatial localization but rather for defining the gaze shift phase (gaze velocity >250 deg/s). Therefore, the analyses in this study focused on relative velocities, amplitudes, and temporal coordination between the eye and head movements.

In the present study, analyses were restricted to the horizontal (yaw-axis) component to specifically examine the characteristics of scanning behavior observed in soccer situations. Furthermore, gaze shift behavior during soccer scanning has primarily been characterized by horizontal exploratory movements (Aksum et al., 2021b; McGuckian et al., 2020; Jordet et al., 2020). Therefore, the gaze shift phase was defined based on the horizontal angular velocity signals of the eye and head movements, and the coordination between these components was analyzed in terms of their amplitude and velocity during that period. Eye angular velocity was calculated by differentiating position data, head angular velocity was obtained directly from the gyroscope, and head position data were calculated by integrating head velocity. Velocity signals were computed from the filtered position data, and all analyses were conducted on the 200 Hz time-series. Figure 2A shows a representative example of the mean positional trajectory during the task, and Fig. 2B presents a representative example of the raw positional trajectory. Note that the eye and head position data represent relative position changes but not absolute positions in space, as mentioned above.

**Figure 2:**
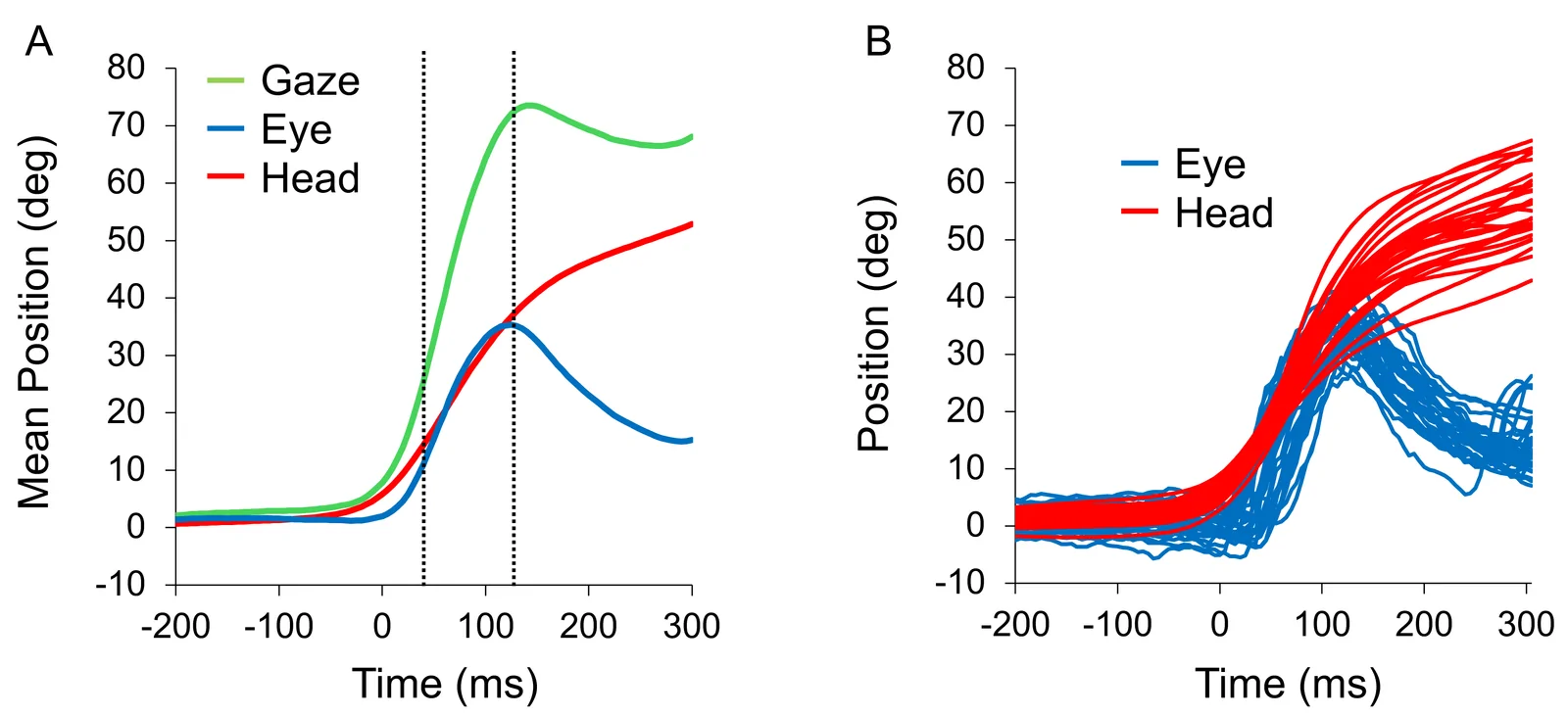
Representative examples of eye and head position traces. (A) Mean waveforms of gaze (green), eye (blue), and head (red) positions. Position traces are plotted as a function of time, with the trial onset position normalized to 0° for both eye and head traces. The vertical dotted lines indicate the gaze shift phase. (B) Representative positional traces from a single trial. Eye (blue) and head (red) positions are plotted as displacements from 0° at trial onset.

In this study, scanning was treated as a conceptual construct following Aksum et al. (2021b), who described scanning as a visual exploratory behavior aimed at situational awareness preceding subsequent play. Previous studies have defined scanning either from observational perspectives using scene recordings or based on velocity thresholds. However, no consistent quantitative standard has been established (Jordet, 2005; McGuckian et al., 2020; Aksum et al., 2021a; Aksum et al., 2021b). In the present study, rather than operationally defining scanning as a whole, we focused specifically on the gaze shift phase as a measurable and fundamental component of scanning behavior. To extract task-relevant epochs from continuous movement data, a head angular velocity threshold (125 deg/s) was used as a practical segmentation and temporal alignment criterion—beginning when horizontal head angular velocity exceeded 125 deg/s and ending when downward vertical head angular velocity exceeded 125 deg/s. Importantly, this threshold was not intended to define scanning itself, nor does it assume that head movement is a necessary or sufficient condition for scanning. Gaze shift phases were independently defined as the period from when gaze angular velocity exceeded 250 deg/s until it fell below this threshold again, and all kinematic variables were calculated within these phases. This threshold was selected as a criterion, assuming practical motion, based on previous studies for head movements (125 deg/s; Chalkley et al., 2018; McGuckian et al., 2019), saccade velocity thresholds (120–150 deg/s; Gregori Grgič, Crespi & De’Sperati, 2016; Kishita, Ueda & Kashino, 2020), and gaze shift velocity thresholds (∼240 deg/s; Parker et al., 2023; Manakhov et al., 2024). By adopting explicit velocity-based criteria for both head and gaze movements, the present study ensures quantitative and reproducible results. The offset criterion was interpreted as an approximate behavioral marker of gaze reorientation from the screen back toward the ball in preparation for passing, rather than a direct indicator of attentional disengagement. Data were segmented from 200 ms relative to this onset until the end of each trial. As participants were instructed to align their head and eye toward the ramp at trial onset, eye position at −200 ms was set as the reference point (0°). Head position was obtained by integrating angular velocity signals; to minimize the effect of integration drift, analyses were restricted to within-trial periods, and head position was normalized to 0° at −200 ms, corresponding to the instructed initial orientation toward the ramp.

Before each trial, all participants were explicitly instructed to fixate on the center of the ramp until the ball became visible. The experimenter continuously monitored adherence to this instruction in real time *via* the Pupil Invisible system. When the infrared sensor at the ball-release point was blocked, the video stimulus was automatically activated, preventing participants from previewing the screen or the target area in advance. To ensure a consistent initial orientation, we applied an objective video-based exclusion criterion. Any trial in which the ball was not visible in the scene-camera frame at the moment it exited the release ramp was excluded. This criterion is theoretically grounded: in scanning research, the disappearance of the ball from the eye-tracking video frame has been used to define the onset of a scan (Aksum et al., 2021a). In other words, having the ball visible in the scene video at that moment is a prerequisite condition for identifying the onset of scanning; if the ball is not visible, it indicates that the participant had pre-rotated the head laterally before the intended gaze shift. Using this objective criterion, 12.1% of all trials were excluded from the analysis. Trial exclusion rates were 13.9% in the 800-ms condition and 10.3% in the 1,600-ms condition (overall: 12.1%), with no systematic bias between conditions. Participant-level exclusions ranged from 0 to 32 trials in the 800-ms condition and 0 to 28 trials in the 1,600-ms condition. This procedure ensured the validity of the straight-ahead fixation assumption (0° normalization) at trial onset and maintained the reliability of gaze and head alignment across all trials. Missing or invalid eye-tracking samples were excluded from analysis; no interpolation or gap-filling procedures were applied.

A 4th-order low-pass Butterworth filter with a cutoff frequency of 50 Hz was applied to reduce measurement noise while preserving the kinematic characteristics of gaze shifts. This cutoff frequency was selected to retain the relevant frequency components of rapid eye movements while attenuating high-frequency noise, consistent with previous studies on eye-movement signal processing (*e.g.*, Mack, Belfanti & Schwarz, 2017). To avoid phase distortion, zero-phase filtering was applied using a forward and reverse filtering procedure. The computed metrics included peak velocity, mean velocity, and amplitude for both eye and head; coefficients of variation (CV); cross-correlations with gaze; and mean-velocity-to-amplitude ratios, as illustrated in Fig. 2. In this study, temporal coordination was defined as the co-modulation of gaze, eye, and head velocity profiles, that is, how their temporal fluctuations evolve together, rather than the temporal offset between their onset times. For this reason, we used the maximum cross-correlation coefficient as the primary metric, as it quantifies waveform similarity and reflects the extent to which the velocity profiles share common control dynamics. In the cross-correlation analysis, both the maximum coefficient and the corresponding lag were calculated for each trial within the gaze shift phase using the actual duration of each phase, and the resulting values were averaged across trials for each participant to obtain representative participant-level measures. This procedure allowed us to report both waveform-level coordination and temporal-offset information using a consistent analytical framework, while maintaining our primary focus on the coordination of velocity profiles.

### Statistics

Paired *t*-tests were used to compare the eye and head variables described in the Data processing section. Effect sizes (Cohen’s *d* or *r*) were also calculated. Cross-correlation analyses were performed to examine the temporal relationships between gaze and eye movements and between gaze and head movements (maximum correlation coefficients reported as CC). In addition, Pearson’s correlation analyses were used to assess associations between continuous variables. The consistency of the Pearson correlation result was further confirmed using Spearman rank correlation.

To account for multiple comparisons, Bonferroni corrections were applied within each set of related paired *t*-tests. Specifically, an adjusted significance threshold of *α* = 0.0167(0.05/3) was applied to the three comparisons of peak velocity, mean velocity, and amplitude, and *α* = 0.025(0.05/2) was applied to the two comparisons of CV of peak velocity and CV of amplitude. The cross-correlation comparison and Pearson correlation were each single tests and therefore did not require correction. A one-way repeated-measures analysis of variance (ANOVA) with Bonferroni-adjusted *post hoc* comparisons was conducted to examine differences in head contribution across different gaze amplitudes. When the assumption of sphericity was violated, the Greenhouse–Geisser correction was applied. To clarify the factor structure of this analysis, gaze amplitudes (0–110°) were divided into three data-driven levels using a tertile split: low (0–40°), mid (40–70°), and high (70–110°). This one-way repeated-measures ANOVA tested whether the head contribution systematically increased with larger gaze amplitudes, a phenomenon well established in classical gaze-control models (Freedman, 2008). To examine the effect of temporal constraints on eye–head coordination, peak velocities were subjected to a two-way repeated-measures ANOVA with temporal (800 ms, 1,600 ms) and effector (eye, head) as within-subjects factors. When significant interactions were observed, Bonferroni-adjusted pairwise comparisons were applied to clarify simple effects. For all analyses, the significance level was set at *α* = 0.05. Statistical analyses were conducted using IBM SPSS Statistics version 30 (SPSS Inc., Chicago, IL, USA). Unless otherwise noted, data are presented as mean ± standard deviation (SD). Prior to all parametric analyses, normality assumptions were assessed using the Shapiro–Wilk test. For paired *t*-tests, normality was evaluated on difference scores; for repeated-measures ANOVAs, normality was evaluated on residuals; and for Pearson correlation, normality was evaluated on the variables of interest. No substantial deviations from normality were observed, with the exception of the lag difference between gaze–eye and gaze–head, for which a Wilcoxon signed-rank test was applied. No extreme outliers were identified in the data. The dataset and SPSS syntax files used for all statistical analyses are publicly available at https://doi.org/10.17605/OSF.IO/E96ZX.

## Results

### Comparison between eye and head movements during gaze shifts

Figure 3A illustrates representative examples of peak velocity and amplitude for the eye and head. As shown in Fig. 3B, paired *t*-tests revealed that peak velocity was significantly larger for the eye than for the head (*t*(19) = 18.31, *p* < 0.001, *d* = 4.09). Mean velocity (*t*(19) = 4.77, *p* < 0.001, *d* = 1.07) and amplitude (*t*(19) = 5.97, *p* < 0.001, *d* = 1.34) were also significantly greater for the eye than for the head. Figure 4A presents representative velocity traces for gaze, eye, and head. As shown in Fig. 4B, cross-correlation analysis revealed that the maximum correlation coefficient (CC) was 0.92 for gaze–eye and 0.58 for gaze–head, with a paired *t*-test indicating that gaze–eye CC was significantly larger (*t*(19) = 30.61, *p* < 0.001, *d* = 6.85). As a supplementary descriptive measure, the temporal lag corresponding to the maximum cross-correlation coefficient was also examined. The mean lag was −0.004 ± 2.89 ms for gaze–eye and 13.12 ± 34.59 ms for gaze–head, where positive values indicate that the head leads the gaze signal. Because the lag variables deviated from normality, a Wilcoxon signed-rank test was conducted, which revealed a significant difference between gaze–eye and gaze–head lag (*Z* =  − 3.883, *p* < 0.001), with gaze–eye exhibiting smaller temporal lag, indicating greater temporal alignment between gaze and eye velocity profiles. These results are interpreted as reflecting stronger temporal coordination and waveform similarity between gaze and eye velocity profiles than between gaze and head velocity profiles, rather than implying a direct causal or hierarchical relationship.

**Figure 3:**
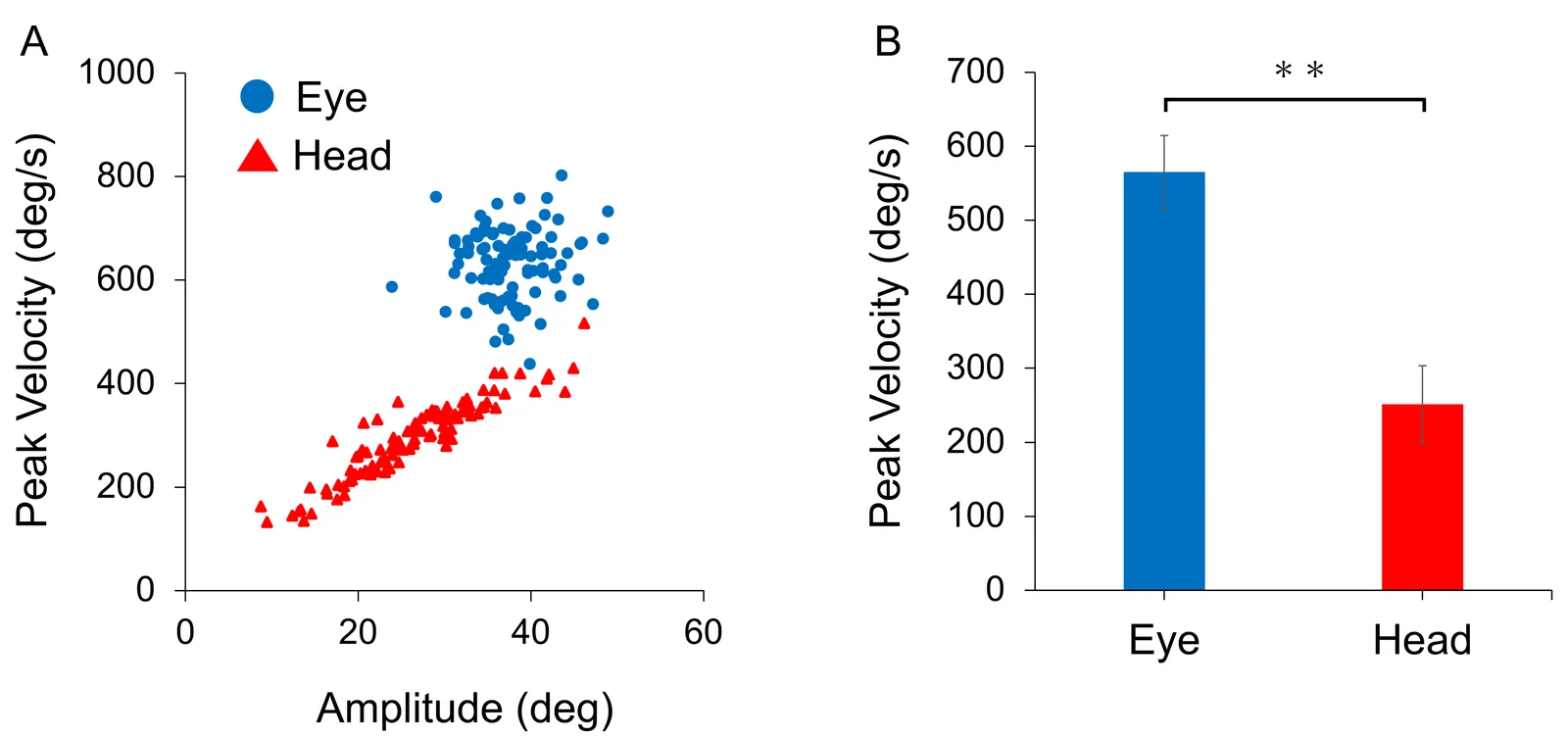
Scatterplots of eye and head peak velocity. (A) Scatterplots of eye (blue) and head (red) peak velocity *versus* amplitude across trials. (B) Comparison of peak eye and head velocity showing larger eye velocity values (**p < 0.001).

**Figure 4:**
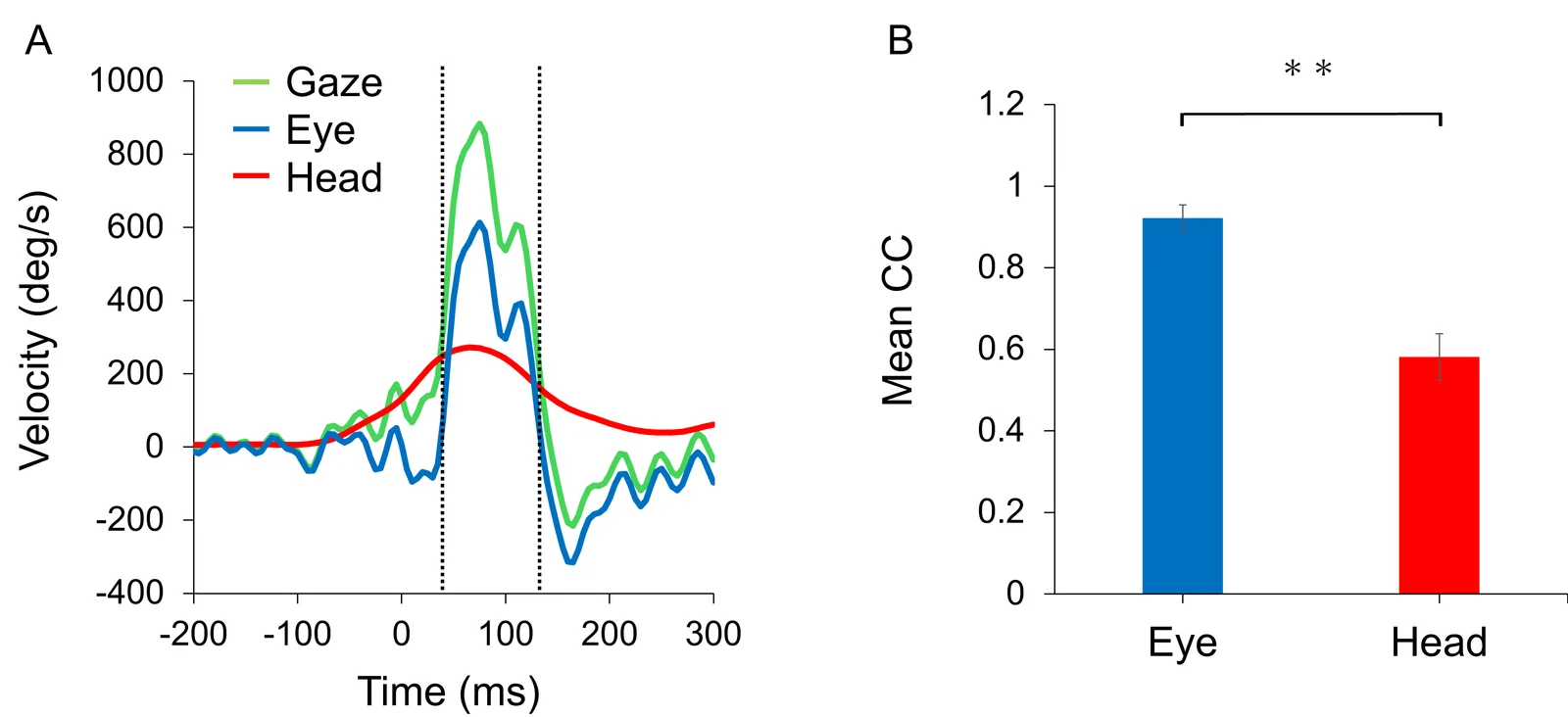
Representative velocity traces. (A) Velocity traces for gaze (green), eye (blue), and head (red). The vertical dotted lines indicate the gaze shift phase. (B) Comparison of maximum cross-correlation (CC) for Gaze–Eye and Gaze–Head (**p < 0.001).

### Relationships between eye and head velocities

Figure 5 shows the mean velocities of the eye and head for each participant. Pearson’s correlation analysis revealed a negative correlation between the two variables (*r* =-0.60, *p* = 0.005). To assess the robustness of this finding, a Spearman rank correlation was additionally conducted, yielding a consistent result (*rs* =-0.567, *p* = 0.009), suggesting that the observed negative correlation is not strongly dependent on outliers. To further assess within-individual consistency of eye–head coordination patterns, Intraclass Correlation Coefficient (ICC) was estimated using a linear mixed model with random intercepts for participants, focusing on the eye-to-head velocity ratio. The result indicated moderate consistency (ICC = 0.44), suggesting that the observed coordination patterns are not attributable purely to random variation. These results suggest that individuals differ in their eye–head coordination tendencies during gaze shifts.

**Figure 5:**
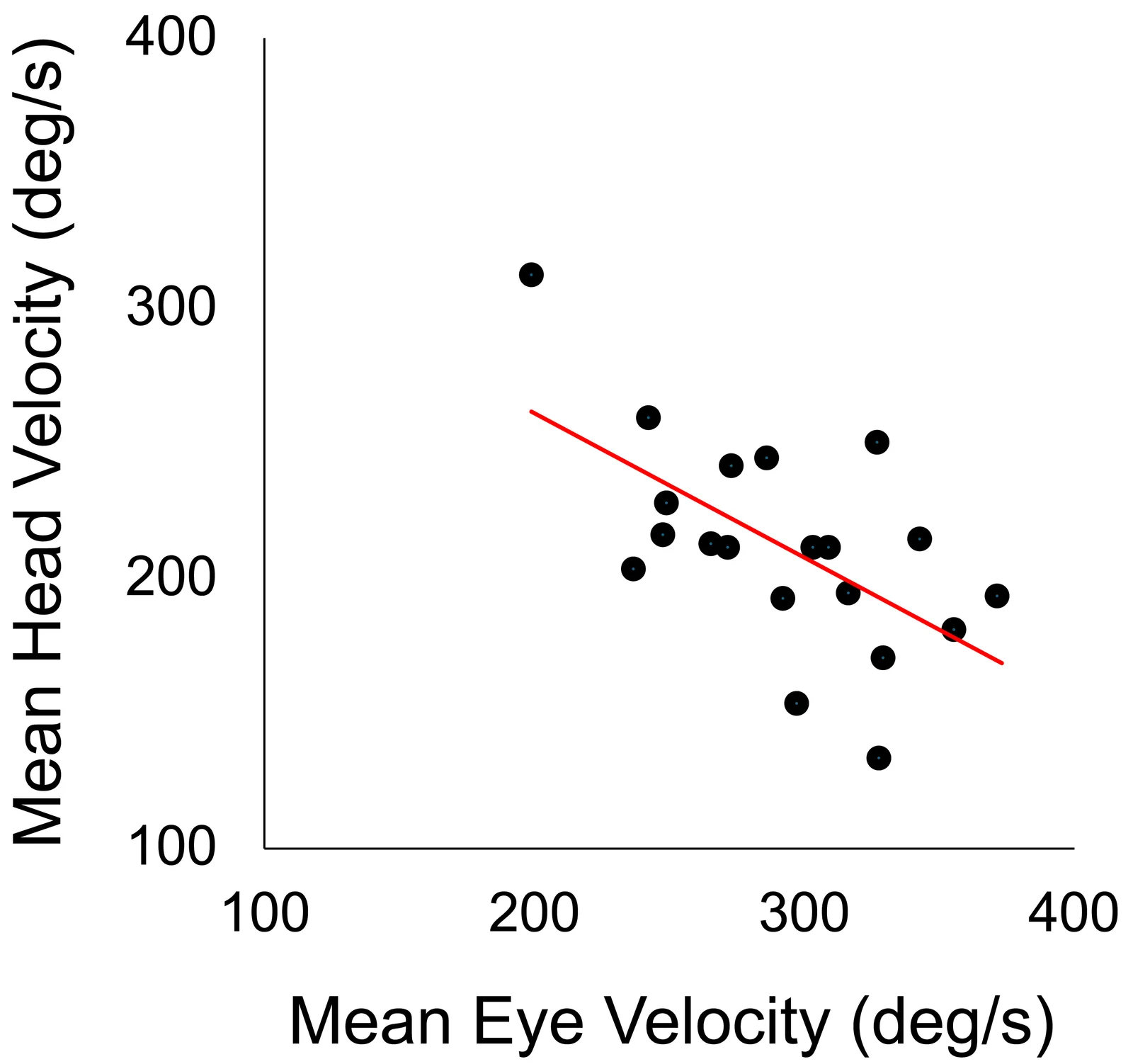
Correlation between eye and head velocities and control profiles. Scatterplot of mean velocity (eye *vs.* head) across participants (*p=0.005).

### Movement stability between eye and head movements

Figure 6A shows the coefficients of variation (CV) of peak velocity for the eye and head across participants. Paired *t*-tests revealed that the CV of peak velocity was significantly smaller for the eye than for the head (*t*(19) = 3.51, *p* = 0.002, *d* = 0.79). Figure 6B displays the CV of amplitude for the eye and head. Paired *t*-tests further showed that the CV of amplitude was also significantly smaller for the eye (*t*(19) = 10.46, *p* < 0.001, *d* = 2.34). In contrast, no significant difference was found for the CV of mean velocity (*t*(19) = 0.76, *p* = 0.457, *d* = 0.17). All significant differences remained below the Bonferroni-adjusted significance thresholds. These results indicate that the eye exhibited lower variability in movement parameters compared with the head. To examine whether head contribution systematically increased as gaze amplitude grew, a one-way repeated-measures ANOVA was conducted with gaze amplitude level as the within-subjects factor. Gaze amplitudes (0–110°) were divided into three data-driven levels using a tertile split: low (0–40°), mid (40–70°), and high (70–110°). Figure 7A shows the changes in head amplitude rate (head amplitude to gaze amplitude) across gaze-amplitude conditions. A one-way repeated-measures ANOVA revealed a significant main effect of gaze amplitude (*F*(2, 28) = 47.80, *p* < 0.001, *ηp*^2^ = 0.773). Bonferroni-adjusted pairwise comparisons showed that the head contribution rate increased significantly from low to mid (*p* < 0.001), mid to high (*p* < 0.001), and low to high (*p* < 0.001) conditions. Figure 7B presents the results for head peak velocity rate (head peak velocity to gaze peak velocity). Mauchly’s test indicated that the assumption of sphericity was violated (*χ*^2^(2) = 8.49, *p* = 0.014); therefore, the Greenhouse–Geisser correction (*ɛ* = 0.676) was applied. The main effect of gaze amplitude remained significant (*F*(1.35, 18.92) = 35.09, *p* < 0.001, *ηp*^2^ = 0.715). Bonferroni-corrected *post hoc* comparisons revealed significant differences between low and mid (*p* < 0.001), low and high (*p* < 0.001), and mid and high (*p* = 0.002) conditions, as illustrated in Fig. 7. These findings indicate that the contribution of head movement systematically increased with greater gaze amplitudes, demonstrating that head involvement becomes more substantial as gaze shifts extend beyond the typical range of eye rotation.

**Figure 6:**
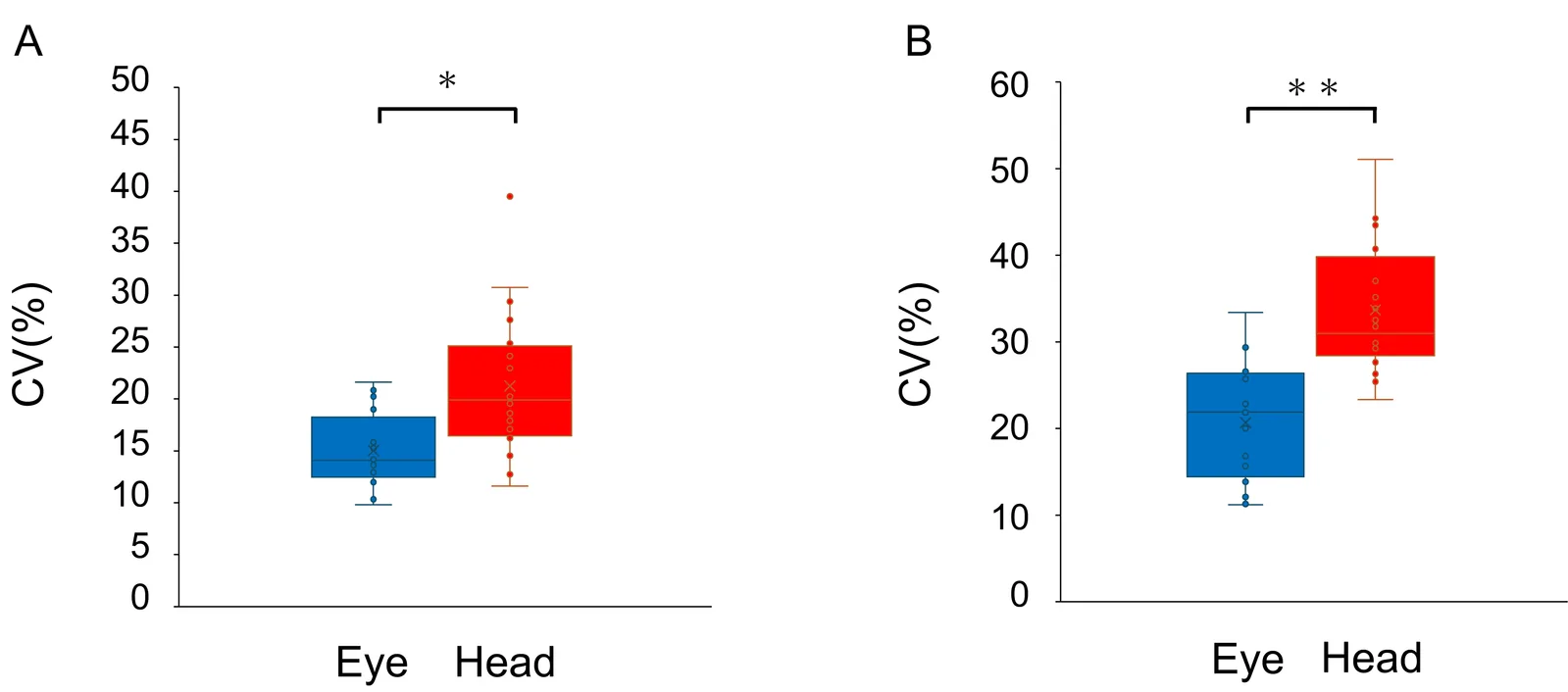
Coefficients of variation (CV) for eye and head. (A) Peak-velocity CV is smaller for the eye (*p = 0.002). (B) Amplitude CV is smaller for the eye (**p < 0.001).

**Figure 7:**
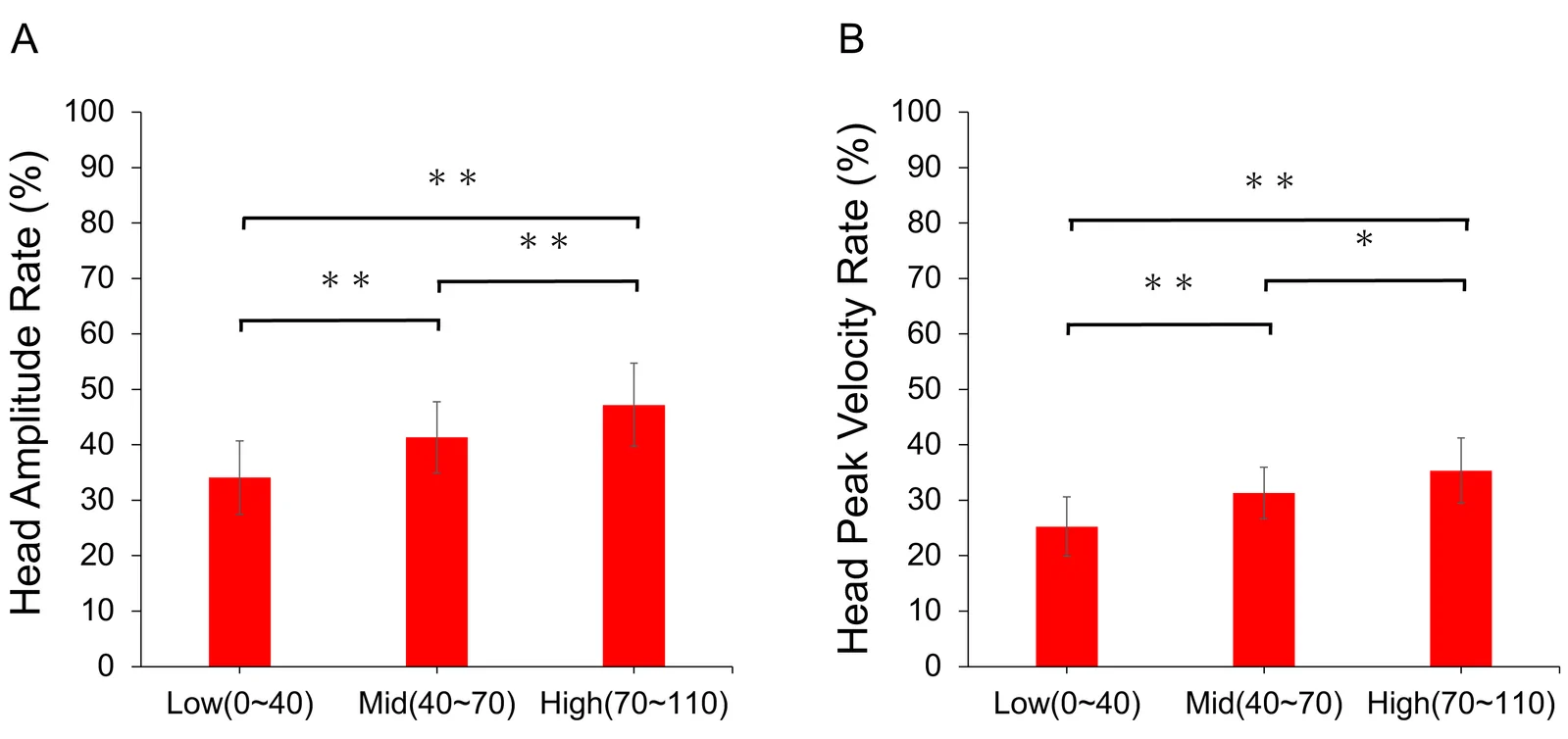
Head contribution across gaze-amplitude conditions. (A) Amplitude Head Contribution Rate increased progressively from Low to Mid to High gaze amplitudes (**p < 0.001, *η*^2^=0.773). (B) Peak Velocity Head Contribution Rate showed the same pattern, with significant differences between all pairs (Low–Mid, Mid–High, Low–High; *p ≤ 0.002; Greenhouse–Geisser *ɛ* = 0.676).

**Figure 8:**
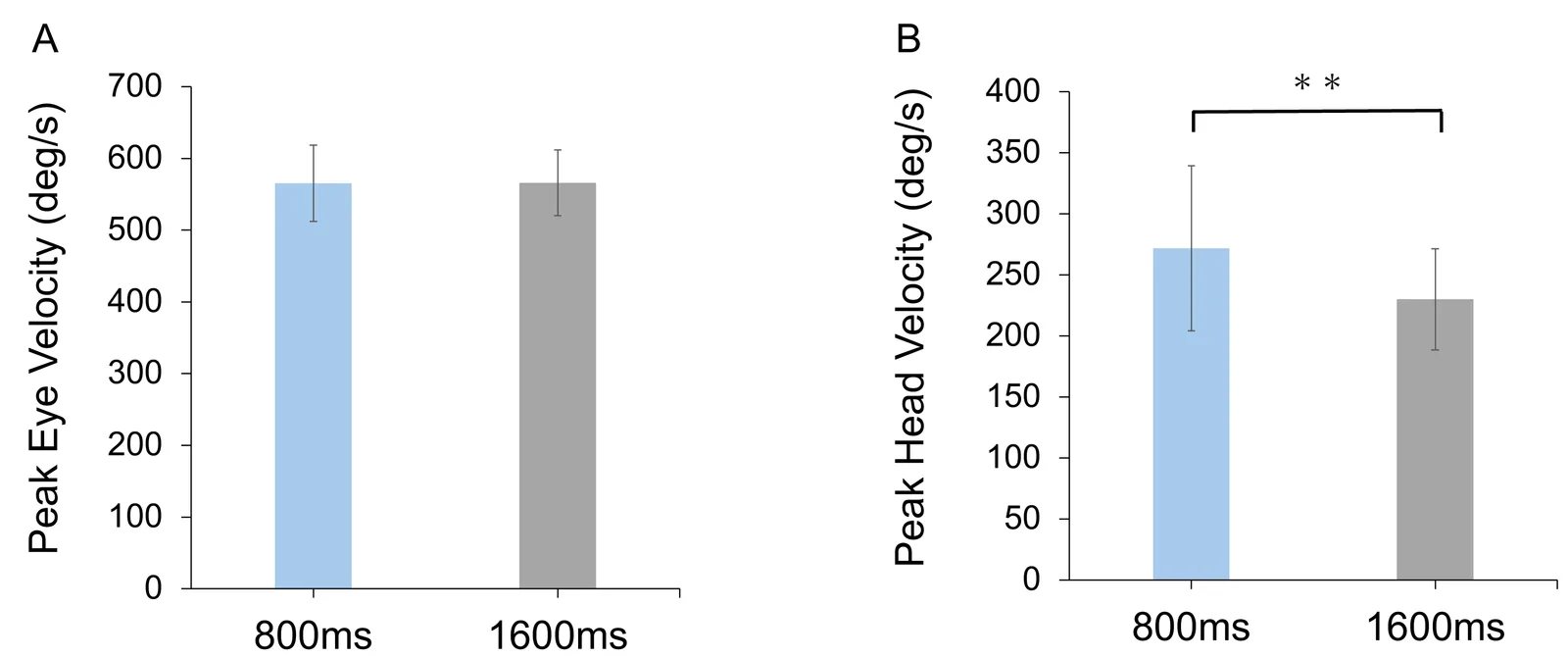
Peak velocity by the temporal conditions (800 ms *vs.* 1,600 ms). (A) Peak eye velocity showed no significant difference between conditions. (B) Peak head velocity was significantly larger under time pressure (800 ms) compared with 1,600 ms (**p < 0.001).

### Difference in eye and head velocities between the temporal conditions

Figure 8 illustrates comparisons of peak velocity under two temporal conditions (800 ms *vs.* 1,600 ms) for the eye (A) and head (B), which served as the two effectors analyzed in this experiment. A two-way repeated-measures ANOVA revealed significant main effects of temporal (*F*(1, 19) = 17.35, *p* < 0.001, *ηp*^2^ = 0.477) and effector (*F*(1, 19) = 344.70, *p* < 0.001, *ηp*^2^ = 0.948). The temporal × effector interaction was also significant (*F*(1, 19) = 20.06, *p* < 0.001, *ηp*^2^ = 0.514). Bonferroni-adjusted pairwise comparisons showed that eye peak velocity did not differ between the 800-ms and 1,600-ms conditions (*p* = 0.898), whereas head peak velocity was significantly larger in the 800-ms condition compared with the 1,600-ms condition (*p* < 0.001). To further characterize these condition effects, amplitude and gaze shift duration were additionally examined. A two-way repeated-measures ANOVA for amplitude revealed significant main effects of Effector (*F*(1, 19) = 35.80, *p* < 0.001, *ηp*^2^ = 0.653) and temporal (*F*(1, 19) = 4.91, *p* = 0.039, *ηp*^2^ = 0.205), as well as a significant temporal × effector interaction (*F*(1, 19) = 11.86, *p* = 0.003, *ηp*^2^ = 0.385). Follow-up simple effects analyses showed that temporal condition had no effect on eye amplitude (*F*(1, 19) = 0.01, *p* = 0.930), whereas head amplitude was significantly larger in the 800-ms condition (*F*(1, 19) = 11.24, *p* = 0.003). Gaze shift duration did not differ significantly between conditions (*t*(19) = 1.50, *p* = 0.150). The 95th percentile values of eye peak velocity were approximately 718 deg/s in the 800-ms condition and 720 deg/s in the 1,600-ms condition, both of which exceeded the reported saccadic saturation range (600–700 deg/s). These findings indicate that while eye peak velocity and amplitude showed no significant differences across temporal conditions, the head showed greater peak velocity and amplitude in the 800-ms condition within the same gaze shift duration, suggesting that the head contributed to extending the spatial range of gaze shifts under time pressure.

## Discussion

### Comparison between eye and head movements during gaze shifts

This study demonstrated that gaze shifts are characterized by a predominant contribution of eye movements relative to head movements. Our results revealed that peak eye velocity and amplitude were significantly greater than those of the head, and the cross-correlation coefficient between gaze and eye was significantly larger than that between gaze and head. These results suggest that gaze velocity profiles are more strongly coordinated with eye movements than with head movements. Previous studies on scanning have primarily focused on the relationship between head movements and performance (Jordet et al., 2020; Berg et al., 2025). However, the contribution of the eye movement has not been sufficiently examined. The novelty of this study lies in the simultaneous measurement of head and eye movements, providing a quantitative characterization of the coordination structure of gaze shifts in the practical context of scanning in soccer. It should be noted that, given the additive model (gaze = eye-in-head + head-in-space), a structural similarity exists between the gaze and eye signals, which may contribute to the higher gaze–eye cross-correlation coefficient. Furthermore, the temporal lag analysis revealed that gaze–eye lag was significantly smaller than gaze–head lag, indicating greater temporal alignment between gaze and eye velocity profiles. These lag results are interpreted as supplementary evidence of waveform similarity rather than evidence of a causal relationship. In the present study, the maximum cross-correlation coefficient was used to quantify how closely the temporal trajectories of gaze, eye, and head velocity evolved together. Therefore, the CC represents the degree to which these velocity profiles co-modulate over time, capturing one aspect of their coordination beyond the magnitude of movement alone. In other words, the eye represents a primary factor shaping the instantaneous form of gaze, reflecting stronger temporal coordination between gaze and eye velocity profiles. By clarifying the predominant contribution of eye movements in scanning, the findings of this study provide an opportunity to reconsider practical scanning instruction, which has traditionally emphasized head turns. Future work may benefit from this type of comprehensive measurement of both eye and head movements, which could lead to more accurate assessments of situational judgments.

### Relationships between eye and head velocities

This study showed that participants differed in their eye–head coordination tendencies during gaze shifts. Pearson’s correlation analyses revealed a negative correlation between the mean eye and head velocities. This negative correlation is consistent with previous research. Earlier studies have shown that as gaze amplitude increases, the contribution of the head motion also increases (Freedman & Sparks, 2000), and that this contribution varies depending on an individual’s gaze shift strategy (Fuller, 1992). The divergence observed in this study, where the contrast between participants with a high contribution of head movement to eye movements and those with the opposite pattern, aligns with these reports.

The superior colliculus (SC) is known to generate shared gaze shift commands for both eye and head movements (Freedman & Sparks, 1997), and therefore, variations in how these commands are distributed across the two effectors likely reflect individual differences in coordination tendencies and motor efficiency within this shared control framework. As a result, the negative correlation between mean eye and head velocities may reflect individual differences in eye–head coordination. It should be noted, however, that this correlation may also arise from differences in overall gaze shift speed or biomechanical scaling properties between eye and head movements, and the present data do not allow us to clearly distinguish between these interpretations. These consistently negative correlations suggested that the relative contribution of eye and head movements varied across individuals, enabling subject-specific coordination profiles to be visualized. This result reflects the variability of individual eye–head coordination tendencies expressed during scanning behavior.

Moreover, these individual scanning behaviors may not solely reflect mechanical movement differences but also visual–perceptual strategies emphasizing efficiency. Even when the eye appears “fixated”, peripheral vision continues to extract critical environmental information that can compensate for limited head or eye displacement. Although visual information uptake is largely suppressed during rapid gaze shifts due to saccadic suppression, the kinematic pattern of this movement may still reflect the efficiency of subsequent visual information acquisition. Therefore, smaller gaze amplitudes do not necessarily imply reduced scanning capability but may instead represent a form of visual economy, an efficient perceptual–motor strategy that minimizes motion while maintaining environmental awareness (Vater, Williams & Hossner, 2020).

Finally, a previous study has reported substantial individual differences in the number of scans performed during actual matches, with disparities exceeding 100 instances (Aksum et al., 2021a). The individual differences in eye–head coordination identified in this study are consistent with such on-field variability, suggesting distinct coordination profiles among players.

### Movement stability between eye and head movements

This study demonstrated that eye movements showed lower variability than head movements during gaze shifts. Our results revealed that the coefficients of variation (CV) for peak velocity and amplitude were significantly smaller for the eye than for the head. In addition, the analysis of head contribution revealed that the proportion of head involvement increased systematically with larger gaze amplitudes. These findings indicate that head contribution to gaze amplitude is nonlinearly related, supporting previous models that the head involvement increases with larger gaze amplitudes (Freedman, 2008; Crawford et al., 1999). The observed peak eye velocity (565 ± 49 deg/s) was close to the saturation range of saccadic velocity (600–700 deg/s; Bahill, Clark & Stark, 1975), and the mean eye amplitude (34.8 ± 6.6°) was near the upper limit of the ocular range of motion (30–40°; Freedman, 2008). Thus, the eye movements were controlled near their physiological limits, resulting in reduced variability in velocity and amplitude and consequently smaller CV values. This lower variability likely reflects the low inertia and reduced antagonist muscle interference of the oculomotor system (Robinson, 1981), allowing the eye to execute superior colliculus (SC) commands with high fidelity. In contrast, the head, which possesses a larger range of motion and greater muscular flexibility without reaching velocity saturation (Zangemeister, Jones & Stark, 1981), compensated for the limitations of the eye motion at larger gaze amplitudes. Indeed, the mean gaze amplitude observed in this study (58.28 ± 9.02°) exceeded the typical ocular range, suggesting that head movement necessarily contributed to accomplishing the task. Therefore, the eye–head coordination observed in this study likely reflects a complementary relationship between the physiological and mechanical constraints of the two effectors. Relatively small gaze amplitudes are primarily executed by the eye, whereas larger gaze shifts recruit the head to compensate for oculomotor limitations. This dynamic interplay allows the gaze control system to maintain both efficiency and flexibility across varying movement demands.

The possibility that consistency of gaze control contributes to the accuracy of decision-making has been noted in reviews of sports science (Hüttermann, Noël & Memmert, 2018). While the present findings reveal individual differences in ocular variability during scanning, the current study did not directly assess its relationship with actual decision-making performance. Previous research has reported that elite soccer players’ superior visual and cognitive functions are associated with executive functions related to decision-making (Knöllner et al., 2022). These findings raise the possibility that ocular variability, a component of the broader visual–cognitive system, may also relate to decision-making processes, although this remains hypothetical. Future research should directly examine whether ocular variability is associated with decision-making accuracy, which may enable the application of these insights to training practice.

### Difference in eye and head velocities between the temporal conditions

Our findings indicated that the head showed greater sensitivity to temporal constraints than the eye, as reflected by increases in both peak velocity and amplitude under the 800-ms condition. The results showed that peak head velocity and amplitude were significantly larger in the 800-ms condition than in the 1,600-ms condition, whereas eye peak velocity and amplitude showed no significant differences across conditions. Gaze shift duration did not differ between conditions, suggesting that faster and larger head movements occurred within the same temporal window under time pressure. This result is consistent with the interpretation that eye movements operate near their physiological limits, as supported by the 95th percentile values of eye peak velocity (approximately 718–720 deg/s across conditions) exceeding the reported saccadic saturation range (600–700 deg/s). Consequently, eye movements showed limited variability in velocity and amplitude, whereas the head showed a greater capacity for adjustment due to the cervical range of motion and muscular flexibility. Indeed, a previous study has shown that peak head velocity continues to increase with amplitude without saturation (Zangemeister, Jones & Stark, 1981), and the present findings are consistent with this. Thus, the findings suggest that under temporal constraints, the contribution of the head increases. Furthermore, it has been reported that temporal pressure alters exploratory behavior in sports contexts (Vater, Williams & Hossner, 2020), which aligns with the present results. From a practical perspective, manipulating time constraints may emphasize the head’s contribution and facilitate training designs that promote broader exploration. For example, time-limited passing tasks based on the present experimental setup have been shown to elicit head-dominant exploration, suggesting potential applications for training. It should be noted that the screen-based setup may limit the direct generalizability of these findings to actual match conditions.

### Limitations and future directions

This study has several limitations. The task was conducted in a controlled, screen-based environment using a 2D video, which differs from real on-pitch conditions in terms of depth cues, vergence, and accommodation, and did not fully reproduce the dynamic uncertainties of actual match play. Foot placement and trunk rotation were not controlled, and trunk co-rotation may potentially influence head-in-space measures.

Methodologically, trial segmentation relied on head movement velocity thresholds, which may not fully capture the functional boundaries of scanning behavior. The downward head movement used as the offset criterion serves as an approximate behavioral marker rather than a direct indicator of attentional disengagement. Complete independence between the segmentation criterion and the analyzed signals cannot be fully guaranteed, although sensitivity analyses using alternative thresholds (200 and 300 deg/s; Data S4 and S5) suggested limited influence on the main findings. The analyses were restricted to horizontal (yaw-axis) movements, and CV is a ratio-based measure whose statistical interpretation should be treated with caution. Integration drift in the gyroscope-derived head position data was not quantitatively assessed. Although drift is expected to be minimal within the short duration of each trial, its effect on the results of this study is likely to be limited.

Regarding sample characteristics, the study was limited to male collegiate soccer players from a single institution in Japan, limiting generalizability across sexes, ages, competitive levels, or cultural contexts. The absence of a control group limits our ability to distinguish general oculomotor characteristics from sport-specific adaptations.

Several analytical directions remain for future research. Controlling for overall gaze velocity or amplitude was considered methodologically difficult given the free-exploration nature of the task. Cluster analysis was not conducted due to the limited sample size (*N* = 20). Fatigue and learning effects were not directly assessed and cannot be fully excluded.

Future studies should examine whether the identified coordination mechanisms generalize to more dynamic, game-like conditions, extend the analysis to three-dimensional eye–head coordination, and include more diverse populations and controlled experimental designs with comparison groups.

## Conclusions

This study aimed to empirically elucidate the structure of eye–head coordination during gaze shifts in soccer scanning, a foundational component of scanning that has not been systematically examined in applied sport contexts. Although mechanisms of eye–head coordination have been extensively characterized in basic sensorimotor research, it has remained unclear whether these principles generalize to the highly dynamic and cognitively demanding environments characteristic of soccer. Our results revealed a characteristic coordination architecture in which gaze shifts are characterized by a predominant contribution of eye movements, while head movements provide task-dependent adjustments to the overall gaze behavior. Furthermore, the coordination pattern systematically varied with contextual demands: head contribution increased with larger gaze-shift amplitudes and under higher temporal constraints. These findings indicate that eye–head coordination is not fixed but instead represents a flexible, multi-structural system that reorganizes according to the spatial and temporal requirements of the task. In addition, the identification of trade-offs in eye–head velocity relationships provides a methodological framework for evaluating scanning behavior beyond traditional head-turn indicators. These results offer a refined foundation for understanding the sensorimotor mechanisms underlying gaze-shift kinematics in soccer scanning. It should be noted, however, that these findings are based on male collegiate soccer players from a single institution, and their generalizability to broader match-like scanning contexts and other populations warrants further investigation.

##  Supplemental Information

10.7717/peerj.21779/supp-1 Data S1 Participant-level summary statistics for gaze, eye, and head kinematics across all trials (gaze velocity threshold: 250 deg/s)

10.7717/peerj.21779/supp-2 Data S2 Participant-level kinematic data separated by temporal condition (800 ms and 1600 ms)

10.7717/peerj.21779/supp-3 Data S3 Participant-level head contribution data across gaze amplitude categories (Low, Mid, High) includes participants with sufficient trials in all categories.

10.7717/peerj.21779/supp-4 Data S4 Sensitivity analysis dataset (gaze velocity threshold: 200 deg/s)

10.7717/peerj.21779/supp-5 Data S5 Sensitivity analysis dataset (gaze velocity threshold: 300 deg/s)

10.7717/peerj.21779/supp-6 Data S6 Trial-level data (long format) for mean eye velocity, mean head velocity, and eye-to-head velocity ratio Used for ICC estimation.

10.7717/peerj.21779/supp-7 Data S7 Summary comparison of key outcomes across sensitivity analysis thresholds

10.7717/peerj.21779/supp-8 Data S8 Eye tracking device

10.7717/peerj.21779/supp-9 Data S9 Experimental overview

10.7717/peerj.21779/supp-10 Supplemental Information 10 Data and code

## Additional Information and Declarations

### Data Availability

The following information was supplied regarding data availability: The raw measurements are available in the Supplemental Files. The data is available at OSF: Saito, Junki. “eye–head Coordination During Rapid Gaze Shifts in Soccer Scanning.” OSF, 5 Apr. 2026. Web. https://doi.org/10.17605/OSF.IO/E96ZX.
