# Supplementary material for: Eye–head coordination during rapid gaze shifts in soccer scanning
Source: PeerJ. 2026 Oct 22;14:e21779. doi: 10.7717/peerj.21779 (PMC13695962; doi:10.7717/peerj.21779)
Supplement: Supplementary file 2 [file peerj-14-21779-s009.pdf]

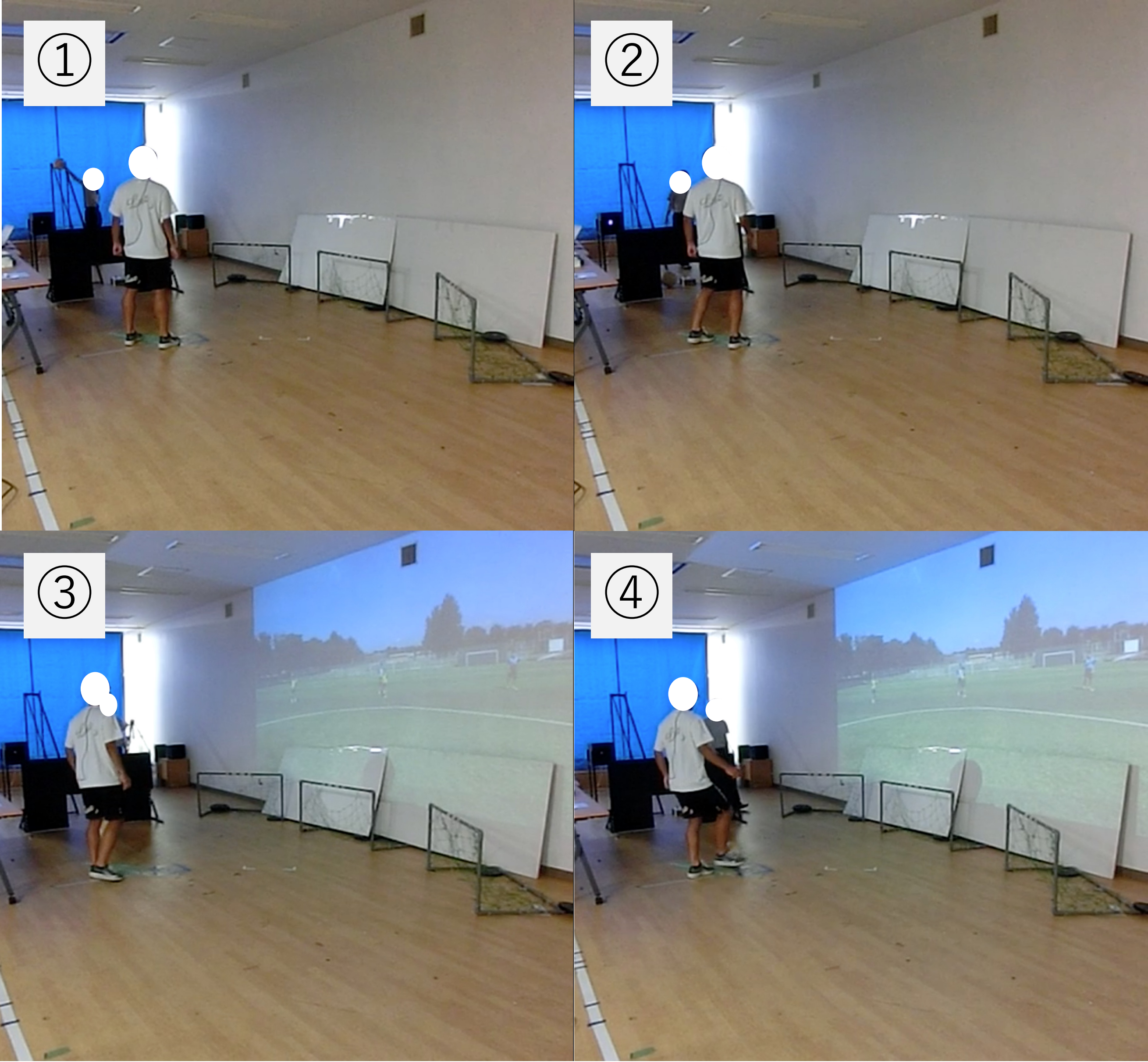
①
②
③
④
